# Advances in HIV Treatment: Long-Acting Antiretrovirals and the Path Toward a Cure

**DOI:** 10.3390/biomedicines13020493

**Published:** 2025-02-17

**Authors:** Massimiliano Lanzafame, Giovanni Mori, Sandro Vento

**Affiliations:** 1 Unit of Infectious Diseases, Santa Chiara Hospital, Azienda Provinciale per i Servizi Sanitari, 38122 Trento, Italy; giovanni.mori@apss.tn.it; 2 Centre for Medical Sciences (CISMed), University of Trento, 38122 Trento, Italy; 3 Faculty of Medicine, University of Puthisastra, Phnom Penh 12211, Cambodia; ventosandro@yahoo.it

Long-acting antiretroviral therapy (LA-ART) represents an important advancement in HIV care as it has considerably reduced the frequency of dosing and therefore improved adherence [1]. The results of several studies conducted among people living with HIV (PLHIV) have highlighted numerous factors that are likely to influence adherence to ART (e.g., medication side effects, stigma, discrimination, and/or non-disclosure of HIV status) [2].

LA-ART formulations, such as injectable cabotegravir and rilpivirine, enable sustained drug release, with dosing intervals of one or two months [3,4,5,6,7,8,9], reducing the risk of patients developing resistance due to fluctuating drug levels. The results of clinical trials such as FLAIR and ATLAS have demonstrated the efficacy of LA-ART in maintaining viral suppression in individuals who were previously on daily oral therapy [4,9]. Furthermore, LA-ARTs improve patient satisfaction, leading to better adherence particularly among those who face stigma or those who do not have regular access to healthcare [10]. In the near future, the development of ultra-long-acting ART (ULA-ART) will extend dosing intervals further, potentially to once every six months or even longer. These therapies, which would include implantable devices and injectable formulations, would offer greater convenience for patients and therefore adherence benefits [11,12,13]. In fact, recent advances in nanotechnology have facilitated the development of long-acting prodrugs and nanoformulations, which could considerably extend the half-life of antiretrovirals [1].

Despite the aforementioned advances, challenges such as injection site reactions and high costs would likely remain, hindering the widespread utilization of LA-ART. Additionally, LA-ART does not address latent viral reservoirs, and the treatment must be continued indefinitely [14]. Therefore, the search for curative therapies continues. Gene therapy, particularly strategies involving CRISPR/Cas9 and other gene-editing technologies, is one approach to eliminate HIV DNA from infected cells, as the results of studies involving animal models have shown that CRISPR/Cas9 can effectively eradicate viral DNA [15,16,17]. Immunotherapies, such as broadly neutralizing antibodies (bNAbs) capable of recognizing a variety of HIV strains, are also being explored to target and eliminate infected cells. These therapies are particularly promising when combined with other strategies, such as latency-reversing agents (LRAs), which aim to “shock” latent HIV out of hiding, making it a target for the immune system [18]. Clinical trials of bNAbs have shown that they can reduce viral load and, in some cases, maintain viral suppression in the absence of ART [19,20,21,22,23]. However, the high production costs involved and the need for frequent dosing are obstacles to their widespread use [18].

Another promising area of research is the combination of different therapeutic approaches to target the HIV reservoir. For instance, combining LA-ART with gene therapy or immunotherapy could reduce the size of the viral reservoir, possibly leading to a functional cure [24]. The concept of a “shock and kill” strategy, where latency-reversing agents are used first to activate dormant HIV, followed by targeted therapies to eliminate infected cells, is a potential pathway toward viral eradication [25]. 

In addition to advancements in therapeutic strategies, public education continues to play a crucial role in the global fight against HIV. Educating people about the modes of HIV transmission, the need for safe practices and regular testing, the correct use of pre-exposure prophylaxis, and the importance of adhering to ART is vital to prevent new infections and improve outcomes. HIV-related stigma is still largely present the world over, and efforts must continue to combat this so that more individuals can seek treatment and adhere to therapies. The continuing advancements in HIV treatment have brought us close to achieving long-term viral suppression and, potentially, a cure. LA-ART has already changed the way we manage HIV, and the development of ultra-long-acting (ULA)-ART, gene therapies, and immunotherapies offers further hope. By combining the latest advances in drug delivery, gene editing, and immunotherapy with a renewed educational effort at the population level, we can move closer to a world where HIV infection will finally be curable.
